# SDH-NET: a South–North-South collaboration to build sustainable research capacities on social determinants of health in low- and middle-income countries

**DOI:** 10.1186/s12961-015-0048-1

**Published:** 2015-10-22

**Authors:** Lucinda Cash-Gibson, German Guerra, V Nelly Salgado-de-Snyder

**Affiliations:** IESE Business School, Barcelona, Ave. Pearson 21, 08034 Barcelona, Spain; National Institute of Public Health, Mexico (INSP), Av. Universidad No. 655 Colonia Santa Maria Ahuacatitlan, CP 62100 Cuernavaca, Morelos Mexico

**Keywords:** Capacity building, Health status disparities, International cooperation, Research, Social determinants of health

## Abstract

**Background:**

It is desirable that health researchers have the ability to conduct research on health equity and contribute to the development of their national health system and policymaking processes. However, in low- and middle-income countries (LMICs), there is a limited capacity to conduct this type of research due to reasons mostly associated with the status of national (health) research systems. Building sustainable research capacity in LMICs through the triangulation of South–North-South (S-N-S) collaborative networks seems to be an effective way to maximize limited national resources to strengthen these capacities. This article describes how a collaborative project (SDH-Net), funded by the European Commission, has successfully designed a study protocol and a S-N-S collaborative network to effectively support research capacity building in LMICs, specifically in the area of social determinants of health (SDH); this project seeks to elaborate on the vital role of global collaborative networks in strengthening this practice.

**Methods:**

The implementation of SDH-Net comprised diverse activities developed in three phases. Phase 1: national level mapping exercises were conducted to assess the needs for SDH capacity building or strengthening in local research systems. Four strategic areas were defined, namely research implementation and system performance, social appropriation of knowledge, institutional and national research infrastructure, and research skills and training/networks. Phase 2: development of tools to address the identified capacity building needs, as well as knowledge management and network strengthening activities. Phase 3: identifying lessons learned in terms of research ethics, and how policies can support the capacity building process in SDH research.

**Results:**

The implementation of the protocol has led the network to design innovative tools for strengthening SDH research capacities, under a successful S-N-S collaboration that included national mapping reports, a global open-access learning platform with tools and resources, ethical guidelines for research, policy recommendations, and academic contributions to the global SDH discourse.

**Conclusions:**

The effective triangulation of S-N-S partnerships can be of high value in building sustainable research capacity in LMICs. If designed appropriately, these multicultural, multi-institutional, and multidisciplinary collaborations can enable southern and northern academics to contextualize global research according to their national realities.

## Background

The process of building sustainable research capacity in low- and middle-income countries (LMICs) is an effective way to overcome limited national resources (financial, human, material) and to pool assets and expertise to ensure that Southern researchers have the full capacity to conduct locally-relevant, high-priority research on public health and health equity, and thus contribute to their own national health system and policymaking process. For this purpose, effective triangulation of South–North-South (S-N-S) partnerships via collaborative research networks can be of high value, as these multi-cultural, multi-institutional and multidisciplinary partnerships can provide an enabling environment for academics (especially for Southern academics) to conduct research in broad areas of public health, as it is contextualized in their national realities and with a deep respect for the idiosyncrasy and identity of each society [1].

In LMICs, there is still a great need for locally-relevant health research evaluations and development of appropriate evidence-based actions in the health field [2]. This is especially the case for research on social determinants of health (SDH), which is the study of “…*the conditions in which people are born, grow, live, work and age. These circumstances are shaped by the distribution of money, power and resources at global, national and local levels*” [3]. This type of research investigates the health disparities across and within societies by examining the socially rooted ‘causes of the causes’ of health problems and inequities at different levels [4, 5].

Given the scope and depth of SDH, research using this approach involves a certain degree of complexity (theoretically, empirically and practically speaking) that requires multi-institutional, multi-disciplinary and, most often, multi-sectoral lenses, as well as methodological innovations that seek to find solutions to specific problems and expand the range of local evidence across thematic areas and country contexts [4]. In other words, the study of SDH calls for a critical mass of researchers from diverse disciplines, with a knowledge of mixed methods to address health inequities through intersectoral actions.

Several calls to rethink existing concepts and methodologies used for the study of SDH have been made by academics in order to make use of global SDH research experiences [6], not being restricted to a Northern perspective, and further integrating interdisciplinary and action-oriented approaches towards solving disparities and local health problems [7, 8]. Such new methods are needed in order to modify SDH, particularly as the traditional categorization of evidence (which places randomized controlled trials and biomedical, laboratory-based experiments at the top of the hierarchy) is not sufficient to develop strategies aimed at promoting health equity, which is the core of SDH [9]. To adequately address community needs under the lens of SDH, it is necessary to analyse daily life conditions, otherwise we run the risk of substantially reducing the scope of knowledge necessary to inform local action [10].

The inclusion of SDH in public policy agendas varies between countries and regions [5], and the overall evidence base and good practices to promote health equity and social justice need to be strengthened world-wide [9]. Moreover, in high-income countries (HICs), there are limited documented experiences regarding successful interventions and implementation approaches to reduce existing health disparities [11]; this supports the need to create S-N-S partnerships that consider global and local perspectives in order to strengthen SDH research capacities.

### SDH research capacity: what is it and how to build it in LMICs?

In many LMICs, there seems to be a limited capacity to conduct scientific research in general, and specifically on SDH, due to a number of reasons, including insufficient research training, scarce financial and material resources, inadequate research output, and the emigration of researchers, often referred to as ‘brain drain’ [12]. Moreover, it has been reported that decision makers in LMICs seldom make effective use of SDH research findings to formulate and implement public policies; as a consequence, public health-related decisions tend to be insufficiently evidence-based [13]. Additionally, the absence of prioritized national health research agendas, integrated within the national health research system, that respond to local needs, negatively affects the interconnection and communication of researchers, policymakers, financing agencies, communities, and other end users of research findings [13, 14]; this leads to failure in adequately translating SDH research into actions, a low production of tailored evidence directed at, and usable by, decision makers [15, 16], and insufficient sharing of this knowledge with the community to support public health literacy. Finally, intersectoral collaborations also need to be strengthened, as many determinants of health lie outside the health sector and need to be addressed under the lens of health in all policies [17].

Given these conditions, research capacity building becomes highly relevant for the international development agenda. It is defined as a process of a very complex nature and “*includes any efforts to increase the ability of individuals and institutions to undertake high-quality research and to engage with the wider community of stakeholders*” [18]. When designing and delivering or implementing capacity building interventions, other factors, such as cultural issues, shifts in power, and system changes, must be considered. Complementing this definition, SDH research capacity building incorporates not only the ability to produce research, but also the capacity to use it and demand it to contribute to health improvement and health equity [12].

The implementation of effective S-N-S SDH research collaborations can play a dynamic role in building and strengthening sustainable SDH research capacity in the various domains of the research system, especially in LMICs. This can be achieved through the creation of local and international SDH networks that build linkages between institutions and stakeholders, establishing new collective perspectives on SDH and health equity. Synergies can be formed to create innovative methodologies and tools to study SDH, and not only to identify, but also to solve, population health needs. This kind of collaboration can also be used for priority-setting and the construction of evidence-based strategies to address SDH and reduce health inequities. Such synergies and solutions may not have been conceived prior to the implementation of the S-N-S partnership, but they emerge as part of an ongoing process [18]. Additionally, effective S-N-S collaborations that include knowledge translation strategies (understood as the exchange, synthesis, and ethical application of the research findings [19]) and social appropriation of knowledge (the process by which scientific SDH knowledge is transferred back to society and used for social growth, development, innovation, and advancement [20]) place special emphasis on the dissemination of key findings to all stakeholders, who may in turn contribute to health equity [12].

Finally, we must mention that traditionally, actions for research capacity building in LMICs are designed using a “top-down” approach, where research is funded and prioritized by external sources, mostly from HICs, with limited participation of LMIC researchers and stakeholders. This dominant approach, however, has been gradually changing to a more inclusive model that encourages the participation of local actors in order to attain a balanced S-N-S collaboration throughout the research process, from the research design to the translation of results and policymaking [21].

### SDH-Net: a South–North-South SDH collaborative Network

The SDH-Net project *SDH-Net: Building Sustainable Research Capacity for Health and its Social Determinants in Low- and Middle-Income Countries* [22] was financed through the European Commission’s 7th Framework Programme (FP7-Contract No. 282534) and is a positive example of research collaboration on SDH for health equity. The design of the study protocol enabled us to find responses to some of the previously highlighted research challenges faced by African and Latin American LMICs, by building sustainable SDH research capacity. SDH-Net consists of a strong and diverse consortium, based on clusters of existing networks of academic institutions from Brazil, Colombia, Mexico, Kenya, South Africa, and Tanzania, in close complementary collaboration with European partners in Spain, Switzerland, the United Kingdom, and Germany. The overall objectives of the SDH-Net project are listed below [22].

- To develop a network for triangulation of knowledge across a S-N-S Collaborative Research Network.
- To build research capacity building tools to generate evidence (on SDH) in each context.
- To build capacity in ethical aspects of SDH research, through the production of useful materials such as guidelines, protocols and tools for implementation.
- To improve knowledge transfer of SDH-related evidence into public policy and action, as well as to address social appropriation of knowledge; specifically to develop policy recommendations to support the translation of SDH research evidence into public policies and highlight how policymakers can and should support the capacity building process in SDH research.
- To strengthen international research networks in order to build sustainable research capacity in SDH.

## Methods

In this section, we describe several methodological aspects to be considered when aiming to build SDH research capacity specifically under the design and implementation of a S-N-S collaboration [23]. Challenges for Southern and Northern partners include responding to key questions such as:

- How can all countries ensure that they equally benefit from the alliances and equally participate in setting the research agenda? (Especially if funding has been secured by Northern collaborators)
- How can the advancement of LMIC research capacity and Southern academics careers be prioritized, while also satisfying the mandates of Northern research institutions?
- How can Southern research institutions coordinate Northern academics’ research efforts on local or regional health issues?

These and many other questions may be posed beforehand, but new ones will certainly emerge throughout the process and several are likely to remain unanswered. Furthermore, Southern academic and research institutions also need to consider and address several elements and challenges when engaging in S-N-S collaborative networks, such as those indicated below and previously elucidated by Chu et al. [23].

Local coordination; Conduct local needs assessments; Address local research capacity building needs; Establish sustainable collaboration (Network Strengthening); Set local research agenda and determine needs for evidence to inform policy and practice; Consider research ethics; Include local authorship and local (as well as international) dissemination of results.

Suggested guiding principles for S-N-S multinational collaborations have also been developed and are indicated below, based on materials by the Swiss Commission for Research Partnerships with Developing Countries [24].

Set the agenda together; Interact with stakeholders; Clarify responsibilities; Account to beneficiaries; Promote mutual learning; Enhance capacities; Share data and networks; Disseminate the results; Pool profits and merits; Apply results; Secure outcomes.

In the case of SDH-Net, the above mentioned principles were embedded in the design of the study protocol and were considered as integral elements throughout the entire project and network development process. In addition, the design of the study protocol allowed for an in-depth and broad SDH capacity-building approach, including managerial and technical aspects, ethical issues, and research strategies, leading to lessons learned on how to build SDH-related research capacity with strong local relevance for all countries involved. The SDH-Net project activities were organized in three phases, as follows:

### Phase 1

The first activities undertaken were to establish an overview of SDH from both the local and global perspectives, and to conduct national mapping exercises to assess the needs of the related research systems through the analysis of its characteristics, stakeholders, institutions, and ongoing SDH activities. Further, current local SDH research capacities were identified within the participating LMICs involved in the project. The examination of the national mapping reports led to the development of a strategic plan to guide the SDH research capacity building approach in Phases 2 and 3 of the project. This strategic plan was based on Diane McIntyre’s conceptual framework, which is cited by Ghaffar et al. [12]. In this framework, five research capacity strengthening dimensions are considered, namely the individual level: analytical and technical skills; the institutional level: organizational research infrastructure such as processes, management, systems, and physical environment; the national research environment: health policy environment, presence of research and academic institutions and organizations; the international research environment: relationship with donors and other international and regional organizations; and research networks: cooperation and information exchange among institutions and organizations. It views capacity strengthening as a long-term, sustainable process which is planned to subsequently create products and activities around a number of action-oriented, high-priority capacity building areas such as (1) research skills and training/networks; (2) institutional and national research infrastructure; (3) research implementation and system performance; and (4) social appropriation of knowledge.

### Phase 2

This phase included the development of research capacity building tools and learning materials to be hosted on an online learning platform. During this phase, there were activities pertaining to networking, internal and external collaboration, staff exchanges, and joint development of a wide range of dissemination activities such as conference presentations, scientific publications, and discussion forums.

### Phase 3

The third and last phase of the SDH-Net project focused on the identification of lessons learned and the development of recommendations in terms of policies and research ethics. Emphasis was placed on how policy and decision-makers can foster the local SDH research capacity building and strengthening process in LMICs.

## Results and discussion

### Local coordination

The Technical and Scientific Coordinators of the SDH-Net project were based in Europe; however, embedded in the project design were two additional lead Regional Coordinators (one in Africa and one in Latin America) whose task was to oversee regional activities. This structure facilitated the South-South triangulation (Africa-Latin America) and South–North-South (Africa-Europe-Latin America) collaborations and ensured that both Southern and Northern institutions were equally engaged in setting and reaching the project objectives, and that Southern institutions could steer the project activities to ensure local relevancy.

### Assessment of local SDH research capacity needs

To guide the direction of the project and its outcomes, assessments of national SDH research capacities were required. This also included the understanding of the processes through which research topics become national and global priorities, how knowledge is produced and translated, and the relationship between stakeholders in the national health research systems. For SDH-Net, this assessment was accomplished when each LMICs used a mapping tool to conduct their national SDH mapping exercise (conducted in 2012, covering the 2007–2012 period). The mapping tool included the description and analysis of (1) SDH research activity; (2) national and global stakeholders in the research environment; (3) national health research systems; and (4) existing SDH research capacities in the country [25]*.*

### Context-specific research capacity building tools – diversifying skill sets

Reports for each LMIC were produced (National SDH Mapping Reports), indicating the specific and contextual needs of SDH research capacities [13, 26]. These national SDH mapping reports were the basis to build the SDH-Net strategic research capacity building plan and subsequent development of innovative SDH research capacity building tools, tailored to fill the gap between the unmet national research priorities and research system needs. This was especially relevant to enable LMIC researchers to conduct high quality SDH research and build diverse research competencies based on their local-regional needs. The areas which needed to be strengthened in order to comprehensively address health equity and associated action research on SDH and health equity are indicated below.

- Social appropriation of knowledge, including awareness of SDH and health inequity issues among policymakers; strengthening of research communication skills; and SDH positioning on strategic political agenda
- Research infrastructure, namely promotion of collaborative networks for research on SDH; enhancing the visibility of (regional) research on SDH; improving incentive mechanisms for publication in national journals; and monitoring of SDH by developing health inequity indicators
- Research capacity, namely strengthening the theoretical, conceptual and methodological basis of SDH (development of SDH content courses and mixed-methodologies used to conduct ‘complex’ SDH research) and strengthening management and research fund management skills and improve SDH research fund management
- SDH Networks, specifically, how to conduct impact evaluation of SDH-related policies and programs and support of regional and inter-regional collaboration in SDH research between SDH-Net partners, as well as with external partners

A unique virtual learning platform was designed to host the developed tools, which included a range of online and classroom-based courses, workshops, case studies, an online case study creator tool, an infographic, methodological guides, and a repository of SDH research-related materials [27]. Accompanying guides were also developed and made available in the platform, which explain how to adapt the resources to diverse modalities, contexts and technical requirements in order to ensure sustainability and use of all deliverables. These guides also describe the pedagogical model used and detail the contents and competencies to be developed with each resource.

**Figure 1:**
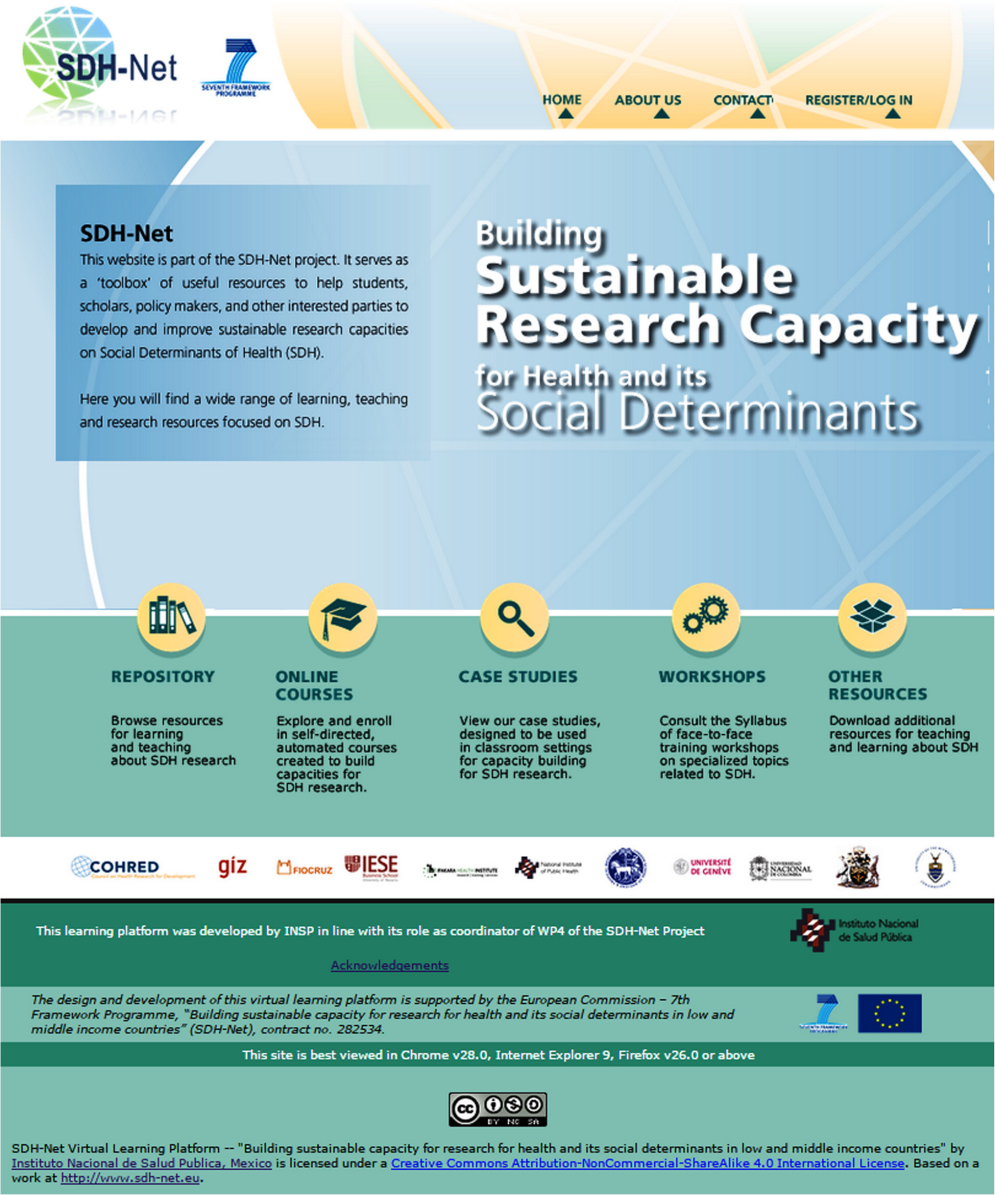
**SDH-Net learning platform: building sustainable research capacity on health and its social determinants.**

In SDH-Net, the innovative research capacity building tools and products reflect the leadership of the local Regional Coordinators and the collective collaboration of Southern and Northern experts on various disciplines relevant to the study of SDH and health equity. Training of Trainers sessions were conducted with consortium members, and learning materials were piloted, adapted, and implemented in participating LMIC institutions and, when possible, aligned with existing curricula. All resources are available under a Creative Commons license and are hosted and freely accessible on the SDH-Net virtual learning platform (Figure 1). These products are conceived as ‘global public goods’ and are being widely disseminated through the National Institute of Public Health (INSP) of Mexico, which has agreed to host and maintain the SDH-Net virtual learning platform for an indefinite period of time. These actions ensure the sustainability of these products beyond the life of the SDH-Net project (2011–2015). Furthermore, INSP secured funds from the Mexican Secretariat of Health to translate most of the products into Spanish, making them available at no cost to all Spanish-language countries interested in building and strengthening SDH research capacities.

### Knowledge management and network strengthening

The establishment of multidisciplinary and multi-institutional S-N-S collaborations can help to overcome the lack of adequate resources, opportunities and infrastructure needed to conduct quality SDH research in LMICs by building and strengthening local collaborative networks. Through this process, Northern and Southern partners can establish fresh perspectives of each other, creating synergies and collaborative innovations in SDH training.

Within SDH-Net, knowledge management and network strengthening were built into the study protocol, subsequently fostered throughout the entire duration of the project. Action plans were developed to support a number of important activities, such as internal and external institutional networking, exchange of researchers, and the joint development of strategies to disseminate findings. Related activities were not only foreseen in the study protocol but were planned into the project budget. For example, SDH-Net institutional exchanges and short-term fellowships, especially for junior researchers, were created to encourage and strengthen cross-sectoral research networking and to utilize and share partner institutions’ expertise. These initiatives are especially important for junior researchers, as they are the next generation of SDH researchers and future agents of change [28].

### Local authorship and national dissemination of results

International South–North authorships in high impact factor scientific journals were encouraged among young researchers from LMICs, to continue their development as scientists in the wider global arena. Furthermore, such authorships and publications were encouraged particularly in open-access scientific journals to ensure their dissemination in LMICs, where academic institutions often lack the financial resources to purchase journal subscriptions or access the pay-per-view publications.

A recurring issue that frequently arises during the development of S-N-S scientific collaboration is that the production of knowledge (in general, but specifically on SDH) rarely reaches audiences at the health policies decision-making sector. This is due to a wide range of causes, but for the Latin-American countries represented in the SDH-Net project, the reason is two-fold. Firstly, there seems to be a lack of expertise and capacities among the scientific community to disseminate their research findings beyond the scientific arena in specialized journals. Nonetheless, when dissemination takes place through scientific journalism, for instance, the national health research system (defined as the people and institutions that generate or use evidence to maintain, promote, and restore the health and development of a population as well as the activities and environment that facilitate these processes [29]) rarely acknowledges their importance for evidence-based policymaking or social appropriation of knowledge. Secondly, some instances of the national health research system tend to privilege the publication of local research findings in foreign language international journals with high impact factor. This situation discourages the national scientific community to publish their findings in local scientific journals, thus hindering the process of knowledge translation into health policies [13].

Another important issue with ethical implications for this type of collaborations is the publication of articles using data collected in LMICs, but lacking local authors. Inclusion, rather than exclusion should be the norm in South–North publications. For instance, in African contexts, where some S-N-S collaborative process are based on “extractive research” as pointed out by Chu et al. [23], they need to ensure that local researchers involved in the process are entitled to authorship when publishing research findings. The SDH-Net project actively promoted collaborations for publications among S-N-S consortium members.

### The importance of knowledge transfer and social appropriation of knowledge: setting local health research agendas

As previously mentioned, many LMICs do not have a prioritized health research agendas integrated within their national health research system. In fact, many countries’ research priorities are still set externally by donors, international organizations and funding agencies outside the national structures, as research is performed where funding money is available [13]. National public policies should reflect national public priorities, and national (health and social) research should be able to adequately address these related concerns and inform decision makers on the creation of evidence-based decisions and strategies directed towards action on SDH and health equity. The failure of policymakers to take up such evidence can depend on the lack of adequate or timely (locally) relevant research, and the accessibility of its findings. Research that answers a policy question at a time when that question is on the political agenda is much more likely to be acted on. Therefore, constructive SDH solutions need to be offered and effectively communicated, moving beyond researcher-centric communication and action paths, and further facilitating the generation, management, translation, and utilization of SDH research [13] by all stakeholders.

SDH-Net has also been designed to facilitate SDH-related knowledge transfer and social appropriation of SDH knowledge, which occurs between the SDH knowledge producers (researcher) and SDH knowledge users (decision makers, society and other researchers). This aims to support the SDH research process (based on community needs) being fed into public policies and practice, and forging stronger links between them.

### The consideration of SDH research ethics

The incorporation of ethical principles in the protocol, particularly in S-N-S research collaborations, was extremely important to prevent different interpretations of ethical standards that may be reflected in failure to properly conduct a study, risking the generation of invalid results that may harm the study. Furthermore, international health equity research has a responsibility to uphold principles of equity, not only through the study itself, but also through the application of ethical criteria to research on SDH [30].

While tackling SDH is considered one of the priorities for the WHO’s general work program during 2014–2019 [31], there are no specific SDH research ethics guidelines, protocols or sections mentioned in international research documents, management or training currently available to support SDH research [30]. SDH-Net also looked into the ethical aspects in SDH research, specifically on how to build capacity in SDH research ethics. A part of the SDH-Net project has involved developing step-by-step global ethical research design guidelines to fulfil this need.

## Conclusions

The development of SDH-Net represents a valuable and innovative experience in the field of SDH research capacity building in LMICs. Reflecting on the design and implementation of the SDH-Net project clearly shows how global initiatives on SDH (seen from the perspective of SDH research) can be effectively put into practice to also strengthen global SDH research capacity (including in HIC settings). What SDH-Net has added to the S-N-S collaborative projects is the fact that it has ventured into addressing SDH research issues not yet tackled even in HIC settings, such as the consideration of SDH research ethics and the development of global ethical research guidelines, or the strengthening of regional SDH networks while preventing “brain drain”, by expanding the local and regional SDH research training opportunities. The innovative SDH-Net learning materials and tools developed are also suitable to meet HIC SDH research needs. Northern research institutions should consider embedding and building on the SDH-Net tools and products developed in their SDH and global health research programs and in public health programs.

As previously discussed, one of the most important purposes of this type of global initiatives is to produce mutual benefit at both global and local levels for all partners involved, but such benefits rarely come beforehand, and are much rather built during the collaborative process itself.

SDH-Net has led us to reflect that, although Northern collaborators can be in a ‘leading’ position derived from their role as a funding source of the project, the constant attitude for discovering, pursuing, and maintaining local concerns on SDH research as a top priority by both Northern and Southern partners – fostered by the distributed coordination of project activities between African, European and Latin American institutions, and a range of South–North and South-South network strengthening activities – can lead to common solutions and new findings not previously considered by both parties during the primary design of the collaboration, thus creating a unique learning experience for all involved.

Consequently, these types of international collaborations can assist in developing solutions to overcome research capacity limitations, further building and strengthening SDH research capacity processes at different levels, both in LMIC and HIC settings. These S-N-S collaborations can also assist in forging sustainable links between different SDH stakeholders – researchers from various disciplines, decision makers, funding agencies, and groups from other sectors within society such as non-governmental organizations; once established, these links can help build sustainable capacity for conducting and managing interdisciplinary SDH research, in HIC and LMIC settings. This, in turn, can increase national autonomy by stimulating scientific excellence and relevance (in SDH and health equity) that support the establishment of research priorities according to local needs.

Based on our experience, it is crucial to ensure that all researchers have the capacity to conduct locally-relevant priority research in the broad areas of public health and health equity, and to be able to contribute to global and national research evidence and thus support local public policy development, with additional efforts and emphases required in LMIC settings. However, all of this can be achieved only if future S-N-S initiatives take into account the need for developing study protocols that embrace the complexity of S-N-S collaborations and capitalize on their full potential in order to reap the mutual benefits and transform them into practical tools for SDH research capacity building.

## Abbreviations

HICs: High-income countries
LMICs: Low- and middle-income countries
SDH: Social determinants of health
SDH-Net: European Commission funded (FP7) collaborative project: SDH-Net: Sustainable Research Capacity for Health and its Social Determinants in Low- and Middle-Income Countries
S-N-S: South–North-South
